# Platelet Function in Stored Heparinised Autologous Blood Is Not Superior to in Patient Platelet Function during Routine Cardiopulmonary Bypass

**DOI:** 10.1371/journal.pone.0033686

**Published:** 2012-03-19

**Authors:** Rolf C. G. Gallandat Huet, Adrianus J. de Vries, Vladimir Cernak, Ton Lisman

**Affiliations:** 1 Department of Anesthesiology, University of Groningen, University Medical Center Groningen, The Netherlands; 2 Surgical Research Laboratory, Dept of Surgery, University of Groningen, University Medical Center Groningen, Groningen, The Netherlands; University of Colorado, United States of America

## Abstract

**Background:**

In cardiac surgery, cardiopulmonary bypass (CPB) and unfractionated heparin have negative effects on blood platelet function. In acute normovolemic haemodilution autologous unfractionated heparinised blood is stored ex-vivo and retransfused at the end of the procedure to reduce (allogeneic) transfusion requirements. In this observational study we assessed whether platelet function is better preserved in ex vivo stored autologous blood compared to platelet function in the patient during CPB.

**Methodology/Principal Finding:**

We measured platelet aggregation responses pre-CPB, 5 min after the start of CPB, at the end of CPB, and after unfractionated heparin reversal, using multiple electrode aggregometry (Multiplate®) with adenosine diphosphate (ADP), thrombin receptor activating peptide (TRAP) and ristocetin activated test cells. We compared blood samples taken from the patient with samples taken from 100 ml ex-vivo stored blood, which we took to mimick blood storage during normovolemic haemodilution. Platelet function declined both in ex-vivo stored blood as well as in blood taken from the patient. At the end of CPB there were no differences in platelet aggregation responses between samples from the ex vivo stored blood and the patient.

**Conclusion/Significance:**

Ex vivo preservation of autologous blood in unfractionated heparin does not seem to be profitable to preserve platelet function.

## Introduction

Cardiac surgery is associated with blood platelet dysfunction and perioperative coagulation disturbances [1], [2]. The use of cardiopulmonary bypass (CPB) (resulting in haemodilution, hemostasis activation by contact with foreign surfaces and tissue factor liberation), blood suction, and the concomitant upregulation of inflammatory pathways all contribute to the activation and mechanical destruction of platelets [3], [4]. Moreover, vasodilatation, and blood loss contribute to a coagulopathic status at the end of surgery. Unfractionated heparin is universally used to anticoagulate patients during CPB, but also to prevent or treat other systemic thrombotic complications. Unfractionated heparin has long been known to possess platelet activating properties, but only recently it has been demonstrated that unfractionated heparin promotes platelet responsiveness via its ability to initiate αIIbβ3-mediated outside-in signaling [5]. During cardiac surgery several techniques are advocated to preserve coagulation factors and platelet function, in order to reduce allogeneic transfusion. These techniques include intra-operative platelet anesthesia [6], [7], platelet-rich plasma harvesting [8] and acute normovolemic haemodilution (ANH) with sequestration of unfractionated heparinised autologous blood [9], [10]. Intraoperative autologous blood can be donated prior heparinisation, after heparinisation but before bypass, and after stabilisation during bypass. In this study, we sequestrated a small volume of autologous blood after heparinisation but prior CPB as is our common practice, to mimick blood storage in the context of normovolemic haemodilution. We hypothesized that platelets function would be better preserved in ex vivo sequestrated unfractionated heparinised autologous blood compared to platelet function in the patient, as platelets in the patient are exposed to other activating mechanisms including the CPB circuit. We measured platelet aggregation responses in cardiac surgical patients and in sequestrated unfractionated heparinised autologous blood during the period of CPB with multiple electrode impedance aggregometry (Multiplate®, Dynabyte, Munchen Germany).

## Methods

### Patients

Twenty-six patients who underwent routine coronary artery bypass grafting (CABG) or valve surgery were included for this observational clinical study after approval of the institutional review board of the University Medical Center Groningen and written informed consent. Patients on aspirin were included, clopidogrel was stopped at least 5 days prior to operation. Excluded were patients who were on unfractionated heparin or intravenous tirofiban prior to surgery, patients with a pre-operative platelet count of <150×10^9^/L or an INR>2.0. Anaesthesia was standardised using bispectral index guided total intravenous anaesthesia with propofol, supplemented by sufentanil (3–4 µg/kg). All patients received tranexamic acid (30 mg/kg). Before CPB, 300 U/kg unfractionated bovine heparin was administered, supplemented if necessary to maintain an activated clotting time of at least 400 sec (ACT, Hemochron®, Technidyne, NY, USA). At the end of CPB, unfractionated heparin was reversed with protamine chloride in a 1∶1 ratio. The open CPB circuit included the use of a membrane oxygenator, roller pumps, uncoated tubings, hyperkalemic cardioplegia (St Thomas solution 2, Plegisol®, Hospira, Lake Forest, Ill, US) and was primed with 1.5 L lactated Ringer's solution and 500 mL 10% Hydroxyethyl starch (Haes 10%, Fresenius®, Bad Homburg Germany). No cell saver was used. The haemostatic profile was measured with a viscoelastic measurement (RoTEM thromboelastometry, Pentapharm®, Munich, Germany). Whole blood coagulation was initiated with tissue factor (ExTEM) without citration and recalcification. Fibrinogen (Clauss) and RoTEM measurements were taken after induction of anaesthesia and after unfractionated heparin reversal.

Blood samples from the patients were drawn gently from an unheparinised 20G arterial line, after discarding 4 times the volume of the connection line. Samples were taken after induction of anaesthesia (pre CPB), 5 min after the start of CPB, at the end of CPB, and after unfractionated heparin reversal.

#### Preparation of autologous blood

After adequate unfractionated heparinisation as assessed with ACT time >400 sec, just before the start of CPB, a sterile blood conservation bag without additives was filled with 100 ml of autologous blood. This procedure was to mimick blood storage during normovolemic haemodilution, but in actual normovolemic haemodilution much larger volumes of blood are stored. This bag was placed on a stirring device (IKA Laboratory Equipment MK 2) and set at 100 cycles/min at room temperature to prevent stasis and spontaneous agglutination of platelets.

Blood samples from the autologous blood bag were taken 5 min after the start of CPB and at the end of CPB. All blood samples taken for platelet function measurements were stored in plastic tubes with hirudin as recommended by the manufacturer (Multiplate®, Dynabyte Medical, Munchen, Germany).

#### Platelet Aggregometry

The Multiplate (Dynabyte® Medical, Munchen, Germany) is a multiple 5 channel whole blood impedance electrode aggregometer with disposable testcells (MEA). The platelet aggregation response was studied with adenosine diphosphate (6.4 µM, ADP), high dose ristocetin (50 uL, 0,77 mg/ml) and thrombin receptor activating peptide (TRAP, 32 µM) a strong activator to assess maximal activation response. ADP and TRAP are direct platelet activators, ristocetin is an indirect activatior which induces von Willebrand factor-mediated platelet activation. The ASPI test with arachidonic acid (0,5 mM), a sensitive platelet aggregation response activator was used to assess aspirin effects on platelet function [11].

#### Statistical Analysis

A decrease in platelet receptor response of 25–50% during CPB has been reported [12]–[14]. A difference of 25% between the platelet aggregation response in the autologous blood bag and in the patient was considered clinically relevant and therefore used for sample size calculation (n = 23, power = 0.8, P<0.05), based on data from the study of Toth [15]. Our data had a normal distribution. Student's t-test for independent samples, was used to assess differences between bag and patient. Repeated Measures Analysis of Variance for multiple comparisons were used to assess a trend over time within the patient. A P-value<0.05 was considered significant. Data were not corrected for haemodilution.

## Results

Blood samples were obtained from 26 patients, 16 patients took aspirin. The demographic data and intraoperative changes in haemostatic profile are shown in table 1. The ADP and TRAP induced intraoperative aggregation response decreased progressively (p^ADP^ = 0.002, p^TRAP^<0.001, for trend over time, table 2). Already in the sample taken 5 minutes after the start of CPB, a significant decline in both ADP- and TRAP-induced platelet aggregation was observed in comparison to aggregation measured in the sample taken after induction of anesthesia (p^ADP^ = 0.004, p^TRAP^ = 0.001). Ristocetin-induced platelet aggregation also declined over time, but the difference in aggregation response between the pre-CPB and 5 minutes after CPB sample was not statistically significant. In samples taken after CPB, ADP- and ristocetin-induced aggregation were lower compared to pre-CPB levels (p^ADP^<0.01, p^RISTO^ = 0.04). In contrast, TRAP-induced aggregation appeared to increase post-CPB, and the post-CPB values were not significantly different from the pre-CPB values (p^TRAP^ = 0.14, table 2).

**Table 1:** Demographic data of the 26 patients studied.

Age (years)	63±15
Gender (m/f)	18/8
CABG/valve surgery/combin(n)	14-10-02
Height (cm)	173±11
Weight (kg)	84±13
CPB period (min)	109±46
Unfractionated heparin U/kg	528±242
Protamine (mg/kg)	5.0±2.4
Chest tube loss 12 hr postoperative (ml)	578±500

	pre CPB	post CPB
Platelet count (×10^9^/L)	209±47	133±35**
Haemoglobin (mmol/L)	7.9±.1.1	4.9±0.5**
Fibrinogen g/L	3.3±1.0	1.9±0.8**
ASPI test (75–136)	49.3±33.9	26.2±26.7**
ExTEM ct (35–80 s)	74.4±14.6	96.0±22.7**
ExTEM cft (35–160 s)	63.4±12.6	93.4±38.7**
ExTEM mcf (53–72 mm)	68.7±6.1	62.0±7.5**
ExTEM α (63–83°)	76.9±3.7	70.1±9.4**

Values are mean ± standard deviation. CPB = cardiopulmonary bypass. CABG = only coronary artery bypass graft, valve = only valve, combin = combination of CABG+valve surgery. ASPI = arachidon acid activated platelet aggregation response, ExTEM = tissue factor initiated whole blood clotting measured with RoTEM (thromboelastometry), ct = clotting time, cft = clot formation time, mcf = maximum clot formation (mm), angle α = indicative for speed of clot generation.

** intraoperative change (p<0.05).

**Table 2:** Platelet activation in patients on CPB and in ex vivo stored unfractionated heparin-anticoagulated blood.

	timepoint	Patient	CI	Blood bag	CI	P
ADP	pre-CPB	59±28	47–70	55±28	44–67	0.86
	5 min CPB	42±23	33–42			
	end CPB	41±21	32–50	45±23	36–55	0.5
	post CPB	39±28	27–50			
TRAP	pre-CPB	103±26	92–113	97±40	81–113	0.53
	5 min CPB	80±35	66–94			
	end CPB	84±46	66–103	83±33	70–97	0.98
	post CPB	92±41	75–108			
Ristocetin	pre-CPB	72±33	59–86	70±38	54–86	0.87
	5 min CPB	64±40	48–81			
	end CPB	54±40	38–71	64±48	44–84	0.43
	post CPB	55±40	38–71			

CPB = cardiopulmonary bypass, ADP, TRAP, Ristocetin = platelet activators, see text. Values represent area under the curve in arbitrarily units (U) given as mean ± standard deviation. CI = 95% confidence interval. P values relate to the difference between samples taken from the patient or from the ex-vivo stored blood at the corresponding time points.

Our study population had reduced platelet aggregation responses in the ASPI test (table 1), which may reflect an existing partial platelet dysfunction in these patients [16]. Nevertheless, preoperative viscoelastic coagulation measurements performed by thromboelastrometry were within normal ranges in all patients.

The measurements of the ADP-, TRAP-, and ristocetin-induced platelet aggregation in the first sample taken from the ex vivo stored autologous blood were not different from the samples taken prior to CPB from the patients (table 2). At the end of the CPB period, the ADP induced platelet aggregation in the ex-vivo stored blood was decreased compared to the first sample taken from the blood bag (p^ADP^ = 0.02, table 2). Decreases in TRAP- and (to a lesser extend) ristocetin-induced platelet aggregation were also observed, but these differences were not statistically significant (p^TRAP^ = 0.08, p^RISTO^ = 0.52). At the end of CPB there was no difference in the platelet aggregation response with all activators between the samples from the ex-vivo stored autologous blood and samples taken from the patient (table 2).

## Discussion

In this observational study we found no difference in the platelet aggregation induced by ADP, TRAP, or ristocetin between samples taken from cardiac surgical patients at the end of CPB and ex-vivo stored heparinised autologous blood. In addition, we found a progressive decrease of ADP- and TRAP-induced platelet aggregation response during CPB, which was already apparent at 5 minutes after the start of CPB. This rapid decrease in platelet aggregation response likely reflects the initiation of CPB, as these findings were not obtained in the sequestrated heparinized blood bag.

Several explanations have been offered for the decrease in platelet function during CPB [3], [4]. The decrease in platelet function in relation to CPB has been demonstrated in many studies in cardiac surgical patients [1], [13], [14], [17]–[20]. These studies are difficult to compare as several methods have been used for the measurement of platelet function, several preservatives were used for storage and sampling and either whole blood or platelet rich plasma was used. Rahe-Meyer [21] also used the Multiplate® to study platelet aggregation during CPB and also found a significant decrease in TRAP- and ADP-induced platelet aggregation, but in this study platelet aggregation was assessed in samples taken 30 min after initiation of CPB. Laga [20] suggested that haemodilution is a major confounder in the measured changes in platelet function during CPB. Recently, an in vitro study in healthy volunteers demonstrated that platelet counts below the normal range due to haemodilution may affect the Multiplate measurements, although even severely diluted samples may give results within the normal range [22]. In our study population, we observed a decrease in platelet count during CPB with a mean platelet count below the normal range. Therefore an effect of haemodilution cannot be excluded. Nevertheless, the decline in platelet function in the patient with decreasing platelet count was not different from the platelet function decline in the ex-vivo stored blood, in which the platelet count was preserved. In our study we did not perform actual isovolemic haemodilution with a clinically relevant volume substitution. Further haemodilution may have obscured the differences caused by unfractionated heparinisation and the artificial surfaces of the storage bag and the cardiopulmonary bypass equipment. Our study was simply designed to mimick the effects of blood storage in the context of normovolemic haemodilution. Another limitation of our study is that the results only pertain to practices where blood is donated prior to haemodilution and bypass. If bypass is started first, blood in the storage bag will be haemodiluted and exposed to the initial damaging effects of CPB. Our study suggests that these effects may not be neglectible. Flom-Halvorsen et al. also assessed the quality of intraoperative autologous blood donation preserved in unfractionated heparin. They found a small increase in beta-thromboglobulin after storage and suggested that heparinised autologous blood was an ideal blood product to restore hemostatic effects [23]. They however did not compare platelet function of the stored blood with platelet function in samples taken from the patients. As we did not measure beta-thromboglobulin we cannot relate our results to their findings. The other preservative widely used for intraoperative autologous blood storage is a citrate containing solution. Citrate, however, functions as an anticoagulant by chelating Ca^2+^ ions. As extracellular calcium is pivotal in platelet activation, this anticoagulant is not advocated for platelet function studies using the Multiplate® [24].

Although it appeared plausible that ex-vivo storage of whole blood in unfractionated heparin would better preserve platelet function as compared to platelet function in the patient, we found no differences in platelet function decline between the two situations. Unfractionated heparin affects platelet function by paradoxical platelet activating properties, which lead to a net decline in platelet activatability [5], [25]–[26]. This decline in platelet functionality is likely caused by irreversible platelet activation by heparin, which may occur in combination with other effects such as platelet activation by the storage bag (see below). When platelets are irreversibly activated, i.e., activated in such a way that granule contents are secreted, they can no longer be activated by a second stimulus, a phenomenon which is well known in literature [27]. Possibly the effects of unfractionated heparin on platelet function as measured by Multiplate® are far more important than the effects of CPB and the surgical procedure on platelet function. Platelet function decline ex vivo may be a combination of the unfractionated heparin effect with effects of the surface of the blood bag (comparable to the surface of CPB tubings) and the temporary storage at room temperature, which may also contribute to platelet activation [28].

In conclusion, we have demonstrated that platelet function is not better preserved in ex-vivo stored autologous blood anticoagulated with unfractionated heparin as compared to platelet function in the patient itself during the same period of cardiopulmonary bypass. Sequestration of autologous blood in unfractionated heparin, as used for acute normovolemic haemodilution in cardiac surgery patients does not seem to be profitable to preserve platelet function.
